# Quality of the record of data on fatal workplace injuries in Brazil

**DOI:** 10.11606/S1518-8787.2017051000064

**Published:** 2017-12-04

**Authors:** Adriana Galdino, Vilma Sousa Santana, Silvia Ferrite

**Affiliations:** I Universidade Estadual do Sudoeste da Bahia. Departamento de Saúde II. Jequié, BA, Brasil; II Universidade Federal da Bahia. Instituto de Saúde Coletiva. Programa Integrado de Pesquisa e Cooperação Técnica em Saúde Ambiental e do Trabalhador. Salvador, BA, Brasil; III Universidade Federal da Bahia. Departamento de Fonoaudiologia. Instituto de Ciências da Saúde. Programa Integrado de Pesquisa e Cooperação Técnica em Saúde Ambiental e do Trabalhador. Salvador, BA, Brasil

**Keywords:** Injuries, Occupational, mortality, Mortality Registries, classification, Data Accuracy, Acidentes de Trabalho, mortalidade, Registros de Mortalidade, classificação, Confiabilidade dos Dados

## Abstract

**OBJECTIVE:**

To evaluate the quality of the data on fatal workplace injuries in Brazil, in the Mortality Information System (SIM) and the Information System of Notifiable Diseases (SINAN-AT), analyzing the spatial and temporal distribution between 2007 and 2012.

**METHODS:**

We identified fields related to fatal workplace injuries, which were examined for completeness and the use of the “ignored” option. From the SIM, we extracted the records of deaths from external causes, which require the completing of the <acidtrab> field about their relation with work. From the SINAN, we analyzed the <evolution> field, which allows us to identify fatal cases among s severe workplace injuries.

**RESULTS:**

In the SIM, from 469,121 records, the <acidtrab> field was left unfilled or filled as ignored in 84.2% of them; the Brazilian region with the highest proportion was the Northeast (79.1%), from which the state of Alagoas (94.4%) had the highest amount. There was a 5.5% decreasing trend between 2007 (86.6%) and 2012 (81.8%). Among the 251,681 records found in the SINAN-AT, 28.3% had unfilled or ignored responses for <evolution>, varying from 39.7% in 2007 to 23.2% in 2012, a 41.6% decrease.

**CONCLUSIONS:**

The quality of the records on the fields of interest needed to identify fatal workplace injuries is poor in the SIM, but gradually improving. Recording quality was better for SINAN-AT, which has also been strongly getting better lately.

## RESUMO

**OBJETIVO:**

Avaliar a qualidade do registro de dados sobre acidentes de trabalho fatais no Brasil nos Sistema de Informação sobre Mortalidade (SIM) e Sistema de Informação de Agravos de Notificação para acidentes de trabalho grave (Sinan-AT), analisando a distribuição espacial e temporal entre 2007 e 2012.

**MÉTODOS:**

Identificaram -se os campos relacionados ao acidente de trabalho fatal, que foram examinados para a completude e uso da opção “ignorado”. Do SIM, foram extraídos os registros de óbitos por causas externas, para os quais é requerido o preenchimento do campo <acidtrab>, sobre a relação com o trabalho. Do Sinan, analisou-se o campo <evolucao>, que permite a identificação dos casos fatais dentre os acidentes de trabalho graves.

**RESULTADOS:**

No SIM, dos 469.121 registros, o campo <acidtrab> foi deixado sem preenchimento ou foi preenchido como ignorado em 84,2%, maior na região Nordeste (79,1%) e, em especial, no estado do Alagoas (94,4%). A tendência foi de queda de 5,5% entre 2007 (86,6%) e 2012 (81,8%). Para os 251.681 registros no Sinan-AT, a falta de preenchimento ou o uso de resposta ignorada no campo <evolucao> representaram, juntos, 28,3%, passando de 39,7% em 2007 para 23,2% em 2012, um declínio de 41,6%.

**CONCLUSÕES:**

A qualidade do preenchimento de campos de interesse para o reconhecimento de casos de acidentes de trabalho fatais é ruim no SIM, mas vem gradualmente melhorando. No Sinan-AT, a qualidade dos registros foi melhor do que no SIM e vem melhorando acentuadamente.

## INTRODUCTION

Fatal workplace injuries (FWA) are one of the health surveillance targets. Therefore, accurate epidemiological estimates are needed, which require good quality records on injuries from the many information systems available. For FWA, data of interest comprise three dimensions: the type of injury, i.e. lesions resulting from injuries; their work-relatedness identification; and whether the outcome was death. Only one-third of the World Health Organization country members had reliable information on workplace injuries in 2000^22^. Underreporting from limited coverage and record quality is a worldwide problem^22^, also found in Brazil^14^. Few studies focus on FWA record quality. A research conducted in the United States has found low accuracy and completeness^16^ in the field for workplace injuries in death certificates. Other studies reported common inconsistencies in the recording fields related to causes of death and their work-relatedness, from information systems^10^ ^,^ ^20^.

In Brazil, the main universal data source of FWA is the Mortality Information System (SIM) based on death certificate data. Despite its advances in coverage and quality, shown by the decrease in ill-defined causes, the SIM is still affected by the poor training of fillers, especially for the coding of causes using the International Classification of Diseases (ICD)^13^ ^,^ ^17^. In the SIM, the relation between the cause of death and work is recorded in the field named “workplace injury”. However, more than three decades after its introduction, the incompleteness of this field is still expressive^2^ ^,^ ^12^ ^,^ ^19^. Another data source of FWA is the Information System of Notifiable Diseases (SINAN), whose subsystem of Severe Workplace Injuries (SINAN-AT), created in 2007, records deaths in the field named “clinical evolution”. Studies on the quality of the SINAN-AT are few and limited to some country areas. In the state of Rio Grande do Norte, in the period of 2007 to 2009, the proportion of incompleteness of the clinical evolution field was 3.9%^6^, much lower than the 24.0% estimated in Betim, state of Minas Gerais, between 2007 and 2011^1^. National estimates and descriptions of spatial distribution and trends over time are lacking. The objective of this study was to estimate measures about the FWA quality of data records, in the SIM and SINAN-AT and to analyze spatial distributions and time changes between 2007 and 2012.

## METHODS

This research was carried out with all deaths from external cause records from the SIM and the work-related injuries from the SINAN-AT, among individuals aged 18 to 65 years, from 2007 to 2012. The SIM database was extracted from the Ministry of Health website, while SINAN-AT data were available at the Collaborating Center for the Surveillance of Workers’ Health, webpage of the Universidade Federal da Bahia. The SIM data analysis focused on work-relatedness, which corresponds to the <acidtrab> field or <evolution> from the SINAN-AT.

The variable injury work-relatedness has the following categories: 1 = yes; 2 = no; or 9 = ignored. In the SINAN-AT, correspondingly, the variable clinical evolution groups are: 1 = cure; 2 = temporary disability; 3 = partial permanent disability; 4 = total permanent disability; 5 = death from work-related injuries; 6 = death from other causes; 7 = other; and 9 = ignored. For both information systems, descriptive variables were calendar year, state or federal district, and region. Data incompleteness was assessed with boxes left unfilled or with ignored answers, analyzed together and separately. These variables were categorized based on quintiles: for the SIM, I (≤ 71.2%), II (71.3 to 77.2%), III (77.3 to 85.8%), IV (85.8 to 90.7%), and V (≥ 90.8%); and for the SINAN-AT, I (≤ 7.5%), II (7.6 to 13.1%), III (13.2 to 16.1%), IV (16.2 to 25.5%), and V (> 25.5%). In addition to absolute and relative frequencies, the percentage proportional variation (PPV) for the time period was calculated. Maps present the spatial distributions. Data were processed in the SAS application, version 9.4. The Project protocol was registered in the National System of Ethics in Research and approved by the Research Ethics Committee of the Instituto de Saúde Coletiva of the Universidade Federal da Bahia (Protocol 927,439, December 15, 2014).

## RESULTS

In the SIM, we found 652,155 records of external cause deaths between 2007 and 2012. Among them, 469,121 (71.9%) had the <acidtrab> field unfilled (Table 1). From the remaining ones 183,034 cases, 80,055 have “ignored” marks, 43.7% of the total. Missing records plus ignored answers amounted to 549,176 (84.2%). Over the study years, the proportion of unfilled fields decreased 8.2%, varying from 75.5% in 2007 to 69.3% in 2012. In contrast, “ignored” answers increased from 11.1% to 12.4%, a PPV = 11.7%. For both, there was a small decline, starting with 86.6% and ending with 81.8%, a 5.5% reduction (Table 1).

**Table 1:** Filling status of the field <acidtrab> in the Mortality Information System (SIM), by calendar year. Brazil, 2007-2012.

Year	Deaths from external causes	Filling status of the field of workplace accident <acidtrab>							
		Unfilled A		Filled 1 = yes or 2 = no B		Filled 9 = ignored C		Unfilled or ignored A+C	
		n	%	n	%	n	%	n	%
Total	652,155	469,121	71.9	102,979	15.8	80,055	12.3	549,1 76	84.2
2007	101,080	76,335	75.5	13,567	13.4	11,178	11.1	87,513	86.6
2008	104,989	77,910	74.2	14,694	14.0	12,385	11.8	90,295	86.0
2009	107,018	78,258	73.1	15,708	14.7	13,052	12.2	91,310	85.3
2010	110,089	77,224	70.1	17,959	16.3	14,906	13.5	92,130	83.7
2011	111,770	78,118	69.9	19,694	17.6	13,958	12.5	92,076	82.4
2012	117,209	81,276	69.3	21,357	18.2	14,576	12.4	95,852	81.8
PPV (2007-2012)			-8.2		35.8		11.7		-5.5

PPV: percentage proportional variation

In Table 2, the amount of unfiled <acidtrab> was expressive, a national average of 71.9%, ranging from 61.4% in the South region to 79.1% in the Northeast; in addition, the highest estimate (94.4%) was for the state of Alagoas while the lowest corresponds to the state of Santa Catarina (49.5%). The estimate of the Midwest region are noticeable as it is below the national average estimate (66.6%), and its low state-specific proportions for Goiás (68.0%), Mato Grosso do Sul (60.9%) and Mato Grosso (57.8%). In 2007, the Northeast (83.5%) held the highest proportion of unfilled <acidtrab>, close to the 2012 estimate of 76.5%. In contrast, the South region had the lowest proportion of this field unfilled, in 2007 (65.0%) and in 2012 (58.6%). Along this time period, there was a slight decrease in the proportion of unfilled <acidtrab> in all regions and most of the country states except for Paraíba, Espírito Santo, and Goiás, in which it increased. The greatest improvement trend in the filing of this field was for the state of Amapá (-17.5%) and Santa Catarina (-17.1%). For both missing data or ignored answers, four states were found in the highest proportion quintile, namely: Alagoas (97.6%), Federal District (94.8%), Rio de Janeiro (92.6%), and Espírito Santo (91.5%). respectively. The lowest quintile, however, were estimated for Tocantins (64.7%), Piauí (69.3%), Roraima (70.4%), and Maranhão (71.1%) (Figure).

**Table 2:** Distribution of records with unfilled <acidtrab> in the Mortality Information System (SIM), by region and federated unit. Brazil, 2007-2012.

Region or Federated Unit		Records with unfilled workplace accident <acidtrab>						
		Total		2007		2012		PPV
		n	%	n	%	n	%	(2007-2012)
Brazil		469,121	71.9	76,335	75.5	81,276	69.3	-8.2
North		38,735	70.5	5,281	72.0	7,102	66.9	-7.1
	Acre	1,496	74.2	223	85.1	297	70.2	-17.5
	Amapá	1,660	75.0	263	79.0	322	75.1	-4.9
	Amazonas	7,656	75.5	976	75.8	1,551	74.2	-2.1
	Pará	19,803	73.4	2,662	74.5	3,408	67.3	-9.7
	Rondônia	4,243	64.3	563	66.5	772	60.5	-9.0
	Roraima	1,083	62.8	182	62.1	188	61.4	-1.1
	Tocantins	2,794	52.8	412	55.5	564	54.9	-1.1
Northeast		157,767	79.1	24,066	83.8	29,004	76.5	-8.7
	Alagoas	15,453	94.4	2,374	95.6	2,667	94.6	-1.0
	Bahia	45,506	83.1	6,584	88.3	8,426	79.3	-10.2
	Ceará	25,037	78.3	3,787	85.7	5,220	75.9	-11.4
	Maranhão	11,922	66.5	1,668	71.6	2,447	65.4	-8.7
	Paraíba	11,224	85.4	1.342	83.0	2.260	88.9	7.1
	Pernambuco	27,597	75.6	5,122	80.0	3,982	69.7	-12.9
	Piauí	6,558	66.5	994	71.8	1,213	61.1	-14.9
	Rio Grande do Norte	8,815	81.7	1,334	86.1	1,640	79.8	-7.3
	Sergipe	5,655	70.0	861	78.8	1,149	73.2	-7.1
Southeast		1 75,583	71.8	31,346	76.0	28,230	68.6	-9.7
	Espírito Santo	12,524	66.5	1,999	64.0	2,099	66.4	3.8
	Minas Gerais	44,127	72.1	7,549	77.7	7,223	65.5	-15.7
	Rio de Janeiro	48,076	81.7	9,429	85.7	7,180	80.4	-6.2
	São Paulo	70,856	67.1	12,369	71.1	11,728	65.2	-8.3
South		59141	61.4	10,012	65.0	9,831	58.6	-9.8
	Paraná	25,494	58.7	4,055	59.3	4,333	56.2	-5.2
	Rio Grande do Sul	23,846	71.9	4,212	77.6	3,977	69.1	-11.0
	Santa Catarina	9,801	49.5	1,745	55.4	1,521	45.9	-17.1
Midwest		10,012	66.6	5,630	67.3	7,109	66.1	-1.8
	Federal District	7,823	81.3	1,210	78.6	1,243	75.2	-4.3
	Goiás	16,269	68.0	2,146	66.2	3,531	71.1	7.4
	Mato Grosso	7,960	57.8	1,261	61.8	1,387	55.5	-10.2
	Mato Grosso do Sul	5,843	60.9	1,013	65.8	948	57.8	-12.2

PPV: percentage proportional variation

**Figure:**
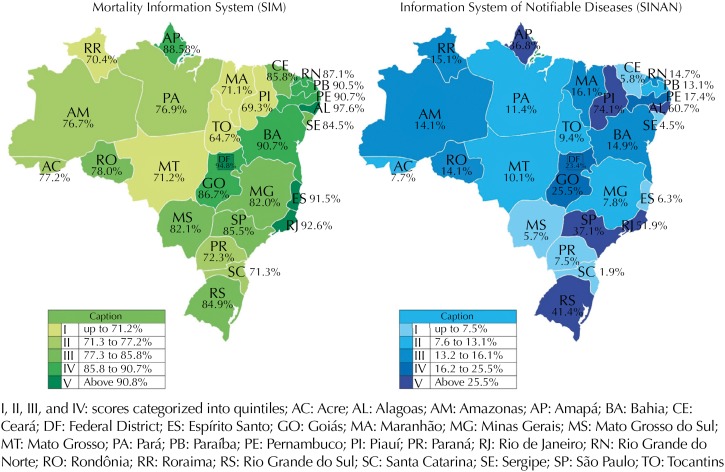
Proportion of records with ignored or unfilled <acidtrab> in the SIM and <evolution> in the SINAN-AT by federated unit. Brazil, 2007-2012.

The SINAN-AT had 251,681 cases reported in the study period (Table 3), from which 16,899 (6.7%) had unfilled <evolucao>. Among the 234,782 cases having this field filled, 54,354 (21.6%) had “ignored” marks. Unfilled plus ignored answers amounted to 71,253 cases (28.3%). The trend over the study time of unfilled <evolucao> boxes ranged from 1,714 (9.0%) in 2007 to 3,030 (4.4%) in 2012, a 51.1% decrease, although the “ignored” answers decreased 38.8%; this is smaller than the declining trend observed for both unfilled and ignored (-41.6%) (Table 3).

**Table 3:** Filing status of the field <evolucao> in the Information System of Notifiable Diseases (SINAN-AT), by calendar year. Brazil, 2007-2012.

Year	Severe workplace injuries	Filing status of the field of evolution of the case <evolucao>							
		Unfilled A		Other types of filling* B		Filled 9 = ignored C		Unfilled or ignored A+C	
		n	%	n	%	n	%	n	%
Total	251,681	16,899	6.7	80,428	71.7	54,354	21.6	71,253	28.3
2007	19,131	1,714	9.0	11,544	60.3	5,873	30.7	7,587	39.7
2008	30,175	2,669	8.8	8,498	61.3	9,008	29.9	11,677	38.7
2009	33,761	2,609	7.7	22,737	67.3	8,415	24.9	11,024	32.7
2010	42,264	4,108	9.7	30,717	72.7	7,439	17.6	11,547	27.3
2011	57,078	2,769	4.8	43,680	76.5	10,629	18.6	13,398	23.4
2012	69,2 72	3,030	4.4	53,252	76.9	12,990	18.8	16,020	23.2
PPV (2007-2012)			-51.1		27.4		-38.8		-41.6

PPV: percentage proportional variation

* 1 = cure; 2 = temporary disability; 3 = partial disability; 4 = permanent total disability; 5 = death from a severe workplace accident; 6 = death from other causes; 7 = another.

The proportion of missing records for evolution varied from 3.8% in the South to 11.2% for the North region (Table 4). Across the country regions and states, the PPV for missing evolution data varies widely. Specifically, between 2007 and 2012, unfiled fields fell from 9.0% to 4.3%, a 51.7% decline. However, it increased in the Northeast and South regions.

**Table 4:** Distribution of records with unfilled evolution of the case <evolucao> in the Information System of Notifiable Diseases (SINAN-AT), by region and federated unit. Brazil, 2007-2012.

Region or Federated Unit		Records with unfilled evolution of the case <evolucao>						
		Total		2007		2012		PPV
		n	%	n	%	n	%	(2007-2012)
Brazil		17,668	6.6	14,775	9.0	3,210	4.3	-51.7
North		1,753	11.2	43	15.2	412	7.9	-48.0
	Acre	25	5.1	0	-	22	7.6	-
	Amapá	1,037	30.1	1	5.0	113	10.7	114.4
	Amazonas	118	5.0	3	23.1	31	4.6	53.8
	Pará	24	3.6	0	-	11	3.3	-
	Rondônia	40	3.3	0	-	18	3.3	-
	Roraima	162	6.0	1	25.0	102	9.1	-63.7
	Tocantins	347	7.4	38	15.8	115	9.4	-75.3
Northeast		1,349	5.1	22	3.8	432	5.0	31.6
	Alagoas	80	8.5	1	20.0	21	6.2	-68.9
	Bahia	3 74	6.5	12	3.6	131	7.9	-33.9
	Ceará	200	3.2	1	1.9	115	4.4	137.8
	Maranhão	243	7.8	0	-	41	3.4	-
	Paraíba	79	4.3	8	6.5	33	7.7	-3.8
	Pernambuco	79	3.2	0	-	35	3.1	-
	Piauí	147	5.9	0	-	3	0.5	-
	Rio Grande do Norte	125	4.8	0	-	50	9.1	-
	Sergipe	22	2,6	0	-	3	1.7	-
Southeast		12,935	7.0	1,672	9.5	1,756	3.9	-58.9
	Espírito Santo	11	3.2	1	14.3	6	4.2	-70.4
	Minas Gerais	792	2.8	13	1.9	314	3.3	75.2
	Rio de Janeiro	380	13.9	89	26.6	75	7.5	-71.9
	São Paulo	11,752	7.7	1,569	9.4	1,361	4.0	-58.1
South		734	3.8	18	2.0	390	4.9	145.0
	Paraná	441	3.1	17	2.2	150	2.7	24.1
	Rio Grande do Sul	2 73	12.3	0	-	232	21.1	-
	Santa Catarina	20	0.7	1	1.4	8	0.7	-52.8
Midwest		897	4.4	20	6.4	220	3.1	-51.6
	Federal District	140	3.1	0	-	0	-	-
	Goiás	344	5.3	1	3.8	49	1.8	-54.4
	Mato Grosso	297	5.8	19	9.5	117	6.7	-64.5
	Mato Grosso do Sul	116	2.8	0	-	54	3.1	-

PPV: percentage proportional variation

Regarding the states, the highest unfiled PPV change over time was estimated for Ceará (137.8%) and Amapá (114.4%). Moreover, reduced missing evolution data was found in Rio de Janeiro (-71.9) and Espírito Santo (-70.4%) (Table 4). Overall, findings for both, unfilled or ignored data show the smallest proportions in Santa Catarina (1.9%) and in Sergipe (4.5%). However, it reached 74.1%, in the state of Piauí (Figure).

## DISCUSSION

In Brazil, between 2007 and 2012, the SIM had an expressive degree of incompleteness of the “work-related injury” field in all regions and federated units, but this has been decreasing, although slowly. In contrast, the proportion of ignored answers showed a slight increase throughout this time. For the SINAN-AT, the overall record quality of the “clinical evolution” field increased, which is a result of the completeness growth while there was a decrease in “ignored” answers. Moreover, the regions with the best SINAN-AT performance were the South and Midwest.

The data quality improvement in relation to FWA, in the SIM, coincides with its system overall advances, such as the coverage increase which reached 96.1% in 2011^9^, and the reduction of non-natural deaths reported as having undetermined intention, which reduced from 10.1% in 2000 to 7.1% in 2011^9^. This contrasts with the persistence of the poor quality in the <acidtrab> filling, which is required to be completed for all external causes of deaths. Presumable explanations are: 1) lack of training of death certificate fillers regarding FWA-related data^17^; 2) poor willing for data recording and limited awareness about the information importance of the work-relatedness registration; 3) lack of infrastructure and personnel to look for additional information needed to determine the relation between work and the injury that cause the death^7^; 4) fear of death certificate fillers, including coroners, about the potential legal consequences of work-relatedness reporting, the police involvement, or law prosecution, when the injury can be framed as a crime^13^.

The high proportions of incompleteness or ignored records found for <acidtrab> in the SIM (national average of 84.2%) are consistent with results from previous studies. For instance, with data from 1997 to 2006, an average 82.9% of external causes deaths had the <acidtrab> filled as ignored^13^; comparable findings have been reported by Santana^19^ for non-filling, 80% on the national average between 2000 and 2010. Distinctively, in the United States, non-filling of the work-relatedness box in death certificates varied from 10% to 30%, across the states^18^.

Examining only the unfilled <acidtrab> in the SIM, high proportions were found in all regions across the country. The worst performance regions and states were those having low income, such as the Northeast, which also have poor performance on health surveillance and other health data^21^. With data from the state of Amazonas, the <acidtrab> field was marked as ignored in 70.0% of the external cause deaths between 2000 and 2011^5^, close to the 75.5% estimated in our study. In this analysis, the state of Tocantins had 52.8% of unfilled “<acidtrab>”, a much lower proportion than the 90.4% estimated in 2004 in another research^2^. Most states had a decrease in incompleteness proportion over their corresponding study times.

In addition, our data on unfiled <acidtrab> need to be interpreted with caution. First, in the electronic form used to feed the SIM, presumably because of the misconception of work-related injuries, the occupational injury option becomes disabled for cases when death is a consequence of violent events^8^ ^,^ ^23^. Thus, even when death certificates are correct, violent-related deaths which could be classified as work-related injuries, are being coded as “homicides,” “suicides,” or “others” This was revealed by the almost total incompleteness of the field <acidtrab> in violent causes of death (99.7%). Narratives from workers in charge of on-line data entry confirm this hypothesis. Work-related injuries do not exclude events involving violence, although it is a common mistake not to recognize their causal relation to work, common in cases of homicides among security personnel.

In the SINAN-AT, the data quality of the “evolution” field was expressive, with high percentages of completeness, although “ignored” answers were common. Over the study time, there was a substantial reduction of unfilled or ignored records. In general, the SINAN data quality is systematically monitored by health surveillance teams, ensuring good performance, notably for death-related data, of great relevance regardless their cause. In addition, the SINAN data are used to end the investigation of notified cases, crucial for evaluation and decision-making in the grounds of epidemiological surveillance^3^. Among all country regions and states, small percentages of unfilled <evolution> fields were estimated. Specifically for the state of Rio Grande do Norte, the 4.8% incompleteness is close to other study findings of 3.9%, from 2007 to 2009^6^. In the city of Betim, state of Minas Gerais, incompleteness of 24.0% between 2007 and 2011 was reported^1^. In our study, for the whole state of Minas Gerais, this was only 3.2%. However, incompleteness decreased over the study time in most regions and states.

We need to keep on mind that the SINAN-AT implementation is still undergoing throughout the country. Although work-related injuries are of compulsory notification since 2004, their data entry was only made possible after 2007 with SINAN. However, according to a 2004 norm, notifications were exclusively reported from sentinel units, i.e., health care units having adequate infrastructure for case identification and reporting, mainly by the Regional Reference Centers for Workers’ Health (CEREST), and specialized hospitals and outpatient facilities. These injury notifications became universal only in 2014. The degree of work-related injury notification was found as “advanced implemented” in only 10 states for 2008^11^. Consistently, only 35.6% of the CEREST considered having reached the status of fully implemented notification of work-related injuries for 2010-2011^15^. In addition, only 28.3% of the total Brazilian municipalities reported work-related injuries in 2011^4^.

The ignored marks in <acidtrab> or in <evolution> must be interpreted considering their operational meaning. The “ignored” code should be chosen when the true response is unknown, and does not represent an error or negligence. The “ignored” answer may be true, reflecting lack of knowledge about the causes of the event of interest, lacking family and colleagues’ information, the inability to carry out visits to workplaces or when the event had occurred for investigation. For <acidtrab>, the overlap between medical and legal aspects may imply failure to provide true responses, thus reducing the filler commitment for accurate recording. The understanding of “ignored” answers as indicating poor data quality is common in studies focusing on the quality of information.

The conclusions of this study should consider its methodological limitations. First, the most important aspect of information quality is the sub-enumeration of cases, which compromises the mortality estimate magnitude. However, in spite of multiple data sources, sub-enumeration was not examined given the difficulties for the identification of all cases, particularly, the lack of a common identifier in the anonymous available databases. Failure to fill <acidtrab>, for example, could be occurring for non-occupational injury cases, although it should be marked as “no”. Unfortunately, this could not be verified given the lack of appropriate data, the same reason why the high SIM field completeness was associated with ignored answers, i.e., lack of knowledge on work-relatedness, despite completion of the field. Regarding SINAN-AT, our conclusions need to take into account the stage of its implementation, not yet completed. The SINAN-AT development involves the training of personnel, often affected by an imbalance between the number of available health workers and the job task demands. Among the limits and challenges faced by the National Network of Integral Care for Workers’ Health (RENAST), the notification of work-related injuries is still under development. For the SINAN-AT, it is also important to consider that the PPV for unfilled boxes presented great variation across states which showed data overdispersion. Specifically, some states had no data for 2007, while in this same year, other states had very small estimates compared to 2012, which contributes to extreme PPV increasing, limiting conclusions.

It is important to notice that both the SIM and SINAN-AT capture work-related injuries for formal and informal workers, the latter excluded from workers’ health statistics until a few years ago, when only workers insured by the Social Insurance were considered.

This study advances the knowledge on the quality of available information systems, a fundamental aspect for workers’ health surveillance. The identification of FWA recording limits can help design strategies to overcome manageable problems. Further studies are recommended focusing on the field filling procedures by directly observing fillers on duty. Research with national estimates of FWA underreporting is also relevant to obtain accurate occupational injury mortality estimates. Our results support the need for consistent investments in the professional training of fillers, the monitoring of the information flow, the data review and analysis, and the dissemination of information, thus improving the quality of FWA data.
